# P-1787. Clinically-Relevant Respiratory Pathogen Detection in Polymicrobial Samples by Targeted Next-Generation Sequencing

**DOI:** 10.1093/ofid/ofaf695.1956

**Published:** 2026-01-11

**Authors:** Sharon K Kuss-Duerkop, Rita Stinnett, Maria C Meriwether, Mikayla Caldwell, Lorraine Abushanab, Emily K DeCurtis, Jena S Tisdale, Robert Schlaberg, Ellie Hasenohr, Yongbao Wang, Reeti Khare

**Affiliations:** National Jewish Health, Denver, Colorado; Illumina, Ogden, Utah; National Jewish Health, Denver, Colorado; National Jewish Health, Denver, Colorado; National Jewish Health, Denver, Colorado; University of Colorado Anschutz Medical Campus, Denver, Colorado; National Jewish Health, Denver, Colorado; Illumina, Ogden, Utah; University of Colorado Anschutz Medical Campus, Denver, Colorado; national jewish health, Denver, Colorado; National Jewish Health, Denver, Colorado

## Abstract

**Background:**

Molecular testing offers a complementary approach to traditional diagnostic methods in complex lung infection cases, for which work-up of non-sterile samples like sputum can be labor-intensive and subject to misinterpretation or missed detections. Targeted next-generation sequencing (tNGS) assays may detect a variety of relevant respiratory pathogens directly from a specimen. The Illumina Respiratory Pathogen ID/AMR Panel Kit, coupled with the DRAGEN Microbial Enrichment Plus application (referred to herein as RPIP; for research use only) can detect and quantify >250 fungi, bacteria and viruses using a targeted hybridization-based enrichment NGS approach. We tested this tNGS assay to evaluate if it could distinguish microbes in diverse, mixed samples.

**Methods:**

More than 120 microbes were divided into polymicrobial pools of 8-15 organisms and spiked into artificial sputum matrix; two pools contained microbes often identified in cystic fibrosis (CF) specimens. Spiked samples underwent DNA and RNA extraction, RPIP preparation, Illumina sequencing and analysis for identification and semi-quantification.

**Results:**

We detected 104/121 (86.0%) expected pathogens, despite being combined in complex pools. Bacteria (68/80, 85.0%) and viruses (21/22, 95.5%) were detected most readily, followed by fungi (15/19, 79.0%). We observed a direct correlation between organism burden (dilution or CFU/mL) and copies/mL, but it differed for each microbe. Detection of cross-reactive organisms occurred in 27/234 (11.5%) samples.

**Conclusion:**

Most microbes were correctly identified from pooled samples, especially CF-associated ones, and included various pathogens, such as *Pseudomonas aeruginosa*, *Mycobacterium avium*, influenza viruses, *Aspergillus fumigatus* and more. Advantages of this tNGS method include high accuracy, wide array of candidate pathogens, identification of rare or fastidious microbes, organism burden quantification from polymicrobial samples, turnaround time and ease of analysis. Limitations include cross-reactivity, susceptibility to contamination and molecular technical expertise. Future directions aim to test the applicability of this tNGS approach in clinical samples, which can often contain various microbes.

**Disclosures:**

Sharon K. Kuss-Duerkop, PhD, Illumina: Grant/Research Support Rita Stinnett, PhD, MHS, Illumina: employee|Illumina: Stocks/Bonds (Public Company) Maria C. Meriwether, BA, Illumina: Grant/Research Support Mikayla Caldwell, MS, Illumina: Grant/Research Support Lorraine Abushanab, PhD, Illumina: Grant/Research Support Robert Schlaberg, MD, MPH, Illumina: IP owned or licensed by Illumina|Illumina: Employee|Illumina: Stocks/Bonds (Public Company) Reeti Khare, PhD, Paratek Pharmaceuticals, Inc.: Advisor/Consultant|Paratek Pharmaceuticals, Inc.: Grant/Research Support

**Figure:**
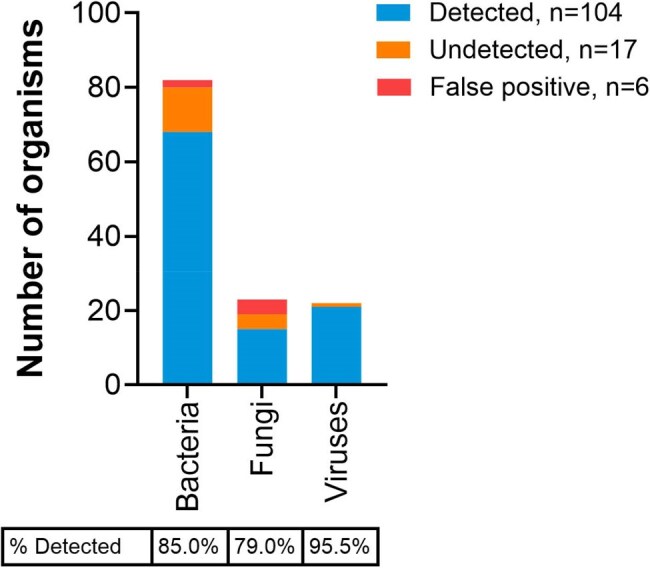
Detection of respiratory organisms from complex pools using a targeted next-generation sequencing (tNGS) method. Respiratory microorganisms were pooled in groups of 8-15 and spiked into artificial sputum matrix for a total of 13 pools. Spiked, artificial sputa underwent nucleic acid extraction, tNGS library preparation and enrichment, and then sequencing. Data shown are detected/undetected organisms from undiluted, spiked pools (121 organisms total).

